# Supplementary material for: Histopathological Growth Patterns Determine the Outcomes of Colorectal Cancer Liver Metastasis Following Liver Resection
Source: Cancers (Basel). 2024 Sep 13;16(18):3148. doi: 10.3390/cancers16183148 (PMC11430747; doi:10.3390/cancers16183148)

Supplementary Table S1: Summary of papers reporting survival analysis of CRCLM based on liver tumor Histopathologic Growth Patterns

Study	Desmopla-stic cut off	Overall survival at 5 years	Median Overall survival (months)	Progression free survival at 5 years	Disease/Recurrence Free Survival	Hazard of death of replacement HGP
Stremitzer *et al.* 2020 [23]	>50%		Not reached dHGP<br>36.6 months rHGP<br>HR 2.32, *p*=0.027		16.3 months dHGP<br>8.7 months rHGP<br>HR 2.6, *p*=0.001	
Galjart *et al.* 2019 [8]	100%	78% dHGP<br>37% non-dHGP<br>HR 0.39,<br>*p* < 0.001	No data	50% dHGP<br>19% non-dHGP<br>HR 0.54, *p* = 0.001	No data	No data
Nierop *et al.* 2019 [7]	100%	No data	No data	6 months<br>71% dHGP<br>57% non-dHGP	17 months dHGP<br>10 months non-dHGP<br>*p* <0.001	No data
Frentzas *et al.* 2016 [6]	>50%	rHGP 21.1%<br>95% CI 5.7-42.9<br>dHGP 51.2%<br>95% CI 21.9-74.5	39.2 rHGP<br>dHGP not reached	No data	No data	No data
Siriwardana *et al.* 2016 [15]	>75%	dHGP 85%<br>95% CI 51-96<br>non-dHPG 54%<br>95% CI 19-70	No data	No data	No data	No data
Eefsen *et al.* 2015 [18]	>75%	**OS worse in rHGP**<br>UVA: HR 1.90,<br>*p*=0.054<br>MVA: HR 2.26,<br>*p*=0.016	No data	No data	**RFS worse in rHGP**<br>UVA: HR 1.93,<br>*p*=0.010<br>MVA: HR 2.16,<br>*p*=0.003	No data
Nielsen *et al.* 2014 [19]	Not given	No data	40.3 dHGP<br>22.8 rHGP<br>Log rank<br>*p*=0.0006	No data	No data	2-2.5x pHGP<br>HR 0.41,<br>p=0.004<br>4x dHGP<br>HR 0.27,<br>p<0.001
Van den Eynden *et al.* 2012 [20]	>75%	**2 yr OS**<br>43.8% pHGP<br>70.2 % rHGP<br>72.5 % dHGP<br>(*p* = 0.02)	No data	No data	No data	No data

Supplementary Table S2: Mutations in the primary tumor and the liver metastases

Supplementary Table S2: Mutations in the Primary Tumor and the Liver Metastases			
	**Desmoplastic N=46**	**Non-desmoplastic N=61**	**P-Value**
**Colorectal tumor mutations**			
KRAS	10 (37.0%)	15 (34.9%)	0.8548
NRAS	2 (7.4%)	1 (2.3%)	0.3069
BRAF	1 (3.7%)	3 (7.0%)	0.5658
Any mutation found in primary tumors	15 (62.5%)	27 (65.8%)	0.785
**Liver tumor mutations**			
KRAS	17 (37.0%)	26 (37.1%)	0.9838
NRAS	2 (4.7%)	0 (0.0%)	0.0687
BRAF	3 (6.7%)	4 (5.7%)	0.8349
TP53	0 (0.0%)	4 (5.7%)	0.1026
APC	2 (4.4%)	9 (12.9%)	0.13440
PIK3CA	2 (4.4%)	8 (11.4%)	0.1945
CTNNB1	0 (0.0%)	1 (1.4%)	0.4207
MAP2K1	1 (2.2%)	0 (0.0%)	0.2103
ERBB2	0 (0.0%)	1 (2.3%)	0.4248
AKT1	1 (2.2)	0 (0.0%)	0.2103
Any mutation found in liver tumors	22 (47.8%)	31 (44.3%)	0.7081
**Total mutations in either liver or colorectal tumor**			
KRAS	25 (39.7%)	34 (38.6%)	0.8966
NRAS	3 (4.9%)	1 (1.1%)	0.1602
BRAF	3 (4.8%)	5 (5.7%)	0.8210
**Number of clinically relevant mutations in the primary tumor**			
0	13 (48.1%)	22 (51.2%)	0.7835
1	11 (40.7%)	14 (32.6%)	
2	3 (11.1%)	6 (14.0%)	
3	0 (0.0%)	1 (2.3%)	
**Number of clinically relevant mutations in the liver tumor**			
0	22 (47.8%)	32 (46.4%)	0.6821
1	18 (39.1%)	23 (33.3%)	
2	6 (13.0%)	13 (18.8%)	
3	0 (0.0%)	1 (1.4%)	

Supplemental Table S3: Summary of mutations based on the location of the primary tumor

Supplemental Table S3: Summary of Mutations based on the location of primary tumor				
	Location of primary tumor			
**Variable**	**Right (N=68)**	**Left/Sigmoid (N=69)**	**Rectum (N= 103)**	**p-value**
**Liver Tumor histology**				
Desmoplastic	31 (46%)	28 (41%)	46 (44%)	0.8261
Non-desmoplastic	37 (54%)	41 (59%)	58 (56%)	
**KRAS mutation in primary tumor**				
Yes	10 (56%)	4 (24%)	11 (34%)	0.1313
No	8 (44%)	13 (76%)	21 (66%)	
**KRAS mutation in liver tumor**				
Yes	19 (56%)	7 (23%)	17 (35%)	**0.0241**
No	15 (44%)	23 (77%)	31 (65%)	
**KRAS mutation in Liver or primary tumor**				
Yes	24 (56%)	9 (25%)	26 (39%)	**0.0197**
No	19 (44%)	27 (75%)	41 (61%)	
**NRAS mutation in primary tumor**				
Yes	1 (6.0%)	2 (12.0%)	0 (0%)	**0.1605**
No	17 (94.0%)	15 (88.0%)	32 (100%)	
**NRAS mutation in Liver tumor**				
Yes	0 (0%)	1 (3.0%)	1 (2.0%)	0.5999
No	33 (100%)	29 (97%)	46 (98%)	
**NRAS mutation in Liver or primary tumor**				
Yes	1 (2.0%)	2 (6.0%)	1 (2.0%)	0.4861
No	41 (98%)	34 (94%)	65 (98%)	
**BRAF mutation in primary tumor**				
Yes	2 (11%)	1 (6.0%)	1 (3.0%)	0.5197
No	16 (89%)	16 (94%)	31 (97%)	
**BRAF mutation in Liver tumor**				
Yes	4 (12%)	1 (3.0%)	2 (3.0%)	0.2581
No	29 (88%)	29 (97%)	46 (96%)	
**BRAF mutation in Liver or primary tumor**				
Yes	5 (12%)	1 (3.0%)	2 (3.0%)	0.0988

No	37 (88%)	35 (97%)	65 (97%)	
**TP53 Mutation in the primary tumor**				
Yes	1 (3.0%)	1 (3.0%)	2 (4%)	0.9601
No	32 (97%)	29 (97%)	44 (96%)	
**APC Mutation in the primary tumor**				
Yes	3 (9.0%)	3 (10%)	4 (8.0%)	0.9690
No	30 (91%)	27 (90%)	44 (92%)	
**PIK3CA Mutation in the primary tumor**				
Yes	7 (21%)	1 (03%)	2 (04%)	**0.0139**
No	26 (79%)	29 (97%)	46 (96%)	
**CTNNB2 Mutation in the primary tumor**				
Yes	1 (03%)	0 (00%)	0 (00%)	0.3034
No	32 (97%)	30 (100%)	48 (100%)	
**MAP2K1 Mutation in the primary tumor**				
Yes	1 (03%)	0 (00%)	0 (00%)	0.3034
No	32 (97%)	30 (100%)	48 (100%)	
**AKT1 Mutation in Liver or primary tumor**				
Yes	1 (03%)	0 (00%)	0 (00%)	0.3034
No	32 (97%)	30 (100%)	48 (100%)	
**ERBB2 Mutation in Liver or primary tumor**				
Yes	0 (00%)	0 (00%)	1 (03%)	0.5740
No	18 (100%)	17 (100%)	31 (97%)	
**Number of Mutation in liver**				
0	9 (26%)	19 (63%)	23 (49%)	**0.0042**
1	12 (35%)	9 (30%)	20 (43%)	
2	12 (35%)	2 (07%)	4 (09%)	
3	1 (03%)	0 (00%)	0 (00%)	
**Number of Mutations in primary tumor**				
0	5 (28%)	10 (59%)	17 (53%)	0.4026
1	9 (50%)	5 (29%)	11 (34%)	
2	3 (17%)	2 (12%)	4 (13%)	
3	1 (06%)	0 (00%)	0 (00%)	

Supplementary Table S4: Summary of exposures vs liver metastasis recurrence

**Supplementary Table S4: Summary of exposures vs. liver metastatic recurrence**				
**Variable**	**Recurrence (N=108)**	**No recurrence (N=99)**	**Never fully resected (N=47)**	**P value**
Age at diagnostic (Mean, SD)	60.60 (±9.73)	62.10 (±10.96)	61.26 (±9.82)	0.1906
BMI (Mean, SD)	26.95 (±5.22)	26.43 (±4.67)	27.97 (±5.38)	0.5996
Volume of primary tumor (Mean, SD)	11.19 (±27.56)	10.53 (±17.09)	9.82 (±13.89)	0.4550
Greatest dimension of liver tumor (Mean, SD)	4.05 (±2.77)	2.94 (±1.90)	3.90 (±3.24)	**0.0037**
Number of liver tumors at first diagnosis (Mean, SD)	3.52 (±2.19)	2.23 (±1.78)	4.74 (±1.85)	**<0.0001**
**Gender**				
Male (N, %)	57 (61%)	73 (63%)	32 (68%)	0.7326
Female (N, %)	36 (39%)	42 (37%)	15 (32%)	
**Liver Tumor histology**				
Desmoplastic (N, %)	46 (40%)	48 (52%)	14 (30%)	**0.0375**
Replacement (N, %)	69 (60%)	45 (48%)	33 (70%)	
**Synchronous presentation**				
Yes (N, %)	78 (68%)	59 (63%)	43 (91%)	**0.0018**
No (N, %)	37 (32%)	34 (37%)	4 (09%)	
**Location of primary tumor**				
Bilateral (N, %)	2 (02%)	4 (04%)	0 (00%)	0.1242
Right (N, %)	26 (24%)	28 (30%)	13 (29%)	
Left (N, %)	33 (30%)	19 (21%)	16 (36%)	
Rectum (N, %)	47 (43%)	40 (43%)	16 (36%)	
**Neoadjuvant chemotherapy before primary resection**				
Yes (N, %)	43 (39%)	23 (26%)	23 (52%)	**0.0120**
No (N, %)	66 (60%)	64 (74%)	21 (48%)	
**Neoadjuvant chemotherapy before liver resection**				
Yes (N, %)	90 (83%)	56 (67%)	40 (91%)	**0.0024**
No (N, %)	19 (17%)	28 (33%)	4 (09%)	

**Neoadjuvant chemotherapy with or without Bevacizumab**				
Neoadjuvent (N, %)	54 (60%)	40 (71%)	19 (48%)	0.0595
BEV (N, %)	36 (40%)	16 (29%)	21 (53%)	
**Development of Pulmonary Metastasis**				
Yes (N, %)	89 (78%)	23 (25%)	35 (80%)	**<0.0001**
No (N, %)	25 (22%)	69 (75%)	9 (20%)	
**Development of Extrahepatic metastatic Disease**				
Yes (N, %)	97 (86%)	27 (29%)	40 (87%)	**<0.0001**
No (N, %)	16 (14%)	65 (71%)	6 (13%)	
**BMI**				
Less than 25	52 (45%)	45 (48%)	18 (38%)	0.7287
Between 25 or 30	37 (32%)	31 (33%)	16 (34%)	
30 and over	26 (23%)	17 (18%)	13 (28%)	

Supplemental Figure S1: Survival of Right-sided Primary tumors by Histopathologic Growth Pattern

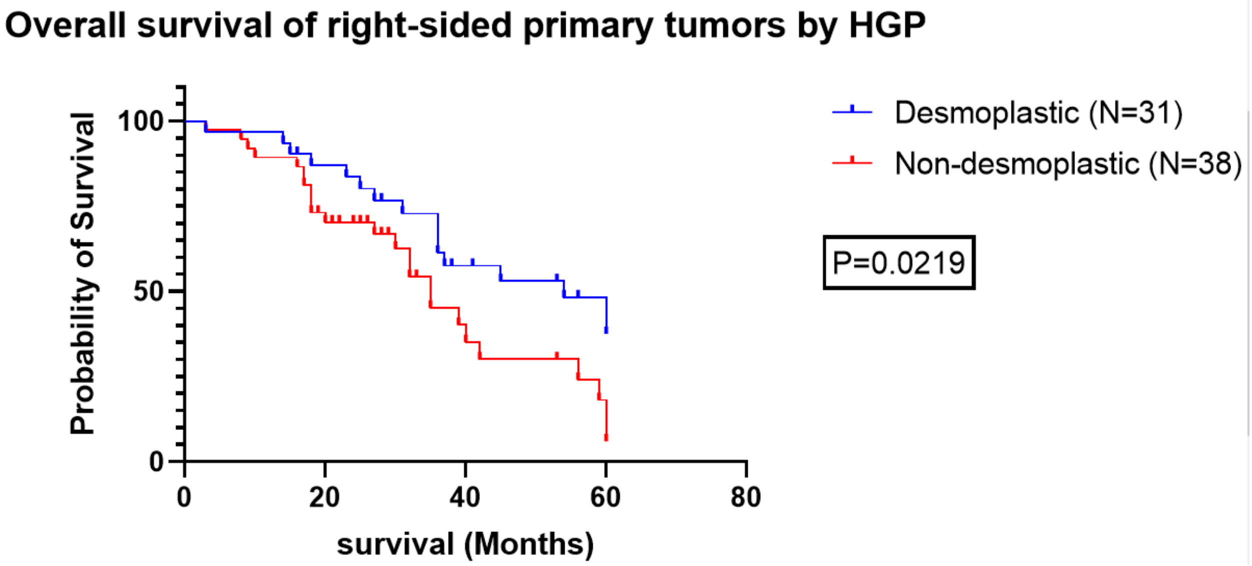

Supplemental Figure S2: Overall survival of entire cohort from diagnosis of liver metastasis

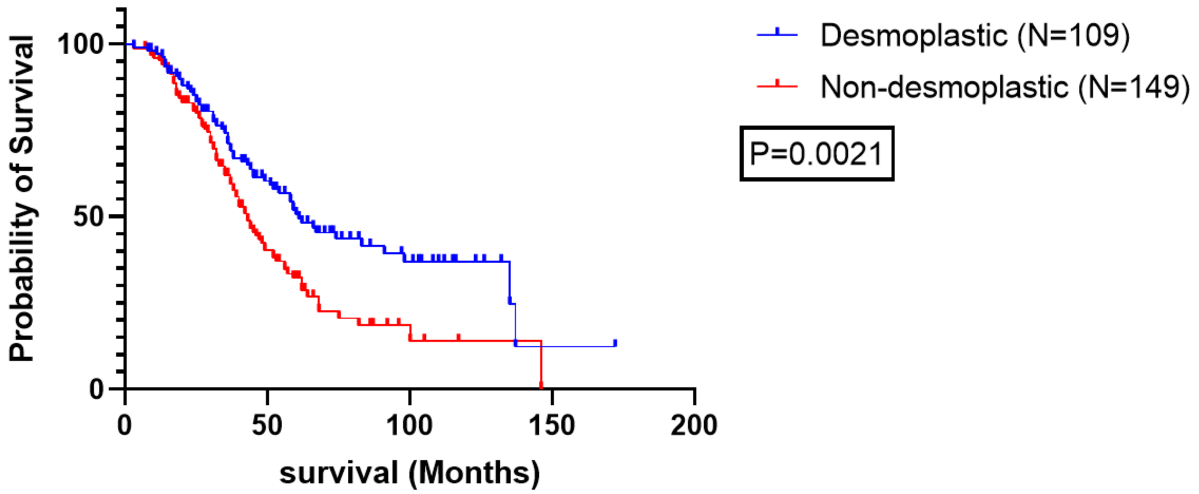

Supplement: Supplementary file 1 [file cancers-16-03148-s001.zip › cancers-3146172-supplementary.pdf]
